# Thrombo-CARE—cardioembolic stroke etiology in cryptogenic stroke suggested by fibrin-/platelet-rich clot histology: Thrombo-CARE (configuration analysis to refine etiology)

**DOI:** 10.1007/s10354-024-01060-w

**Published:** 2024-11-11

**Authors:** Daniel C. Schwarzenhofer, Tim von Oertzen, Serge Weis, Michael Sonnberger, Joachim Gruber, Anna Tröscher, Helga Wagner, Philipp Hermann, Birgit Grubauer, Judith Wagner

**Affiliations:** 1 Department of Neurology, Neuromed Campus, Kepler University Hospital, Wagner-Jauregg-Weg 15, 4020 Linz, Austria; 2 Division of Neuropathology, Neuromed Campus, Department of Pathology and Molecular Pathology, Kepler University Hospital, Johannes Kepler University, Linz, Austria; 3 Clinical Research Institute for Neurosciences, Johannes Kepler University, Linz, Austria; 4 Department of Neuroradiology, Neuromed Campus, Kepler University Hospital, Linz, Austria; 5 Center for Clinical Studies (CCS Linz) and Clinical Research, Johannes Kepler University, Linz, Austria; 6 Department of Neurology, Evangelisches Klinikum Gelsenkirchen, Academic Hospital University Essen-Duisburg, Gelsenkirchen, Germany; 7 Department of Medical Statistics and Biometry, Institute of Applied Statistics, Johannes Kepler University Linz, Linz, Austria

**Keywords:** Thrombectomy, Secondary prevention, Immunohistochemical, CD3, CD45

## Abstract

**Background:**

Despite extensive diagnostic efforts, the etiology of stroke remains unclear in up to 30% of patients. Mechanical thrombectomy (MT) potentially enhances etiological determination by (immuno)histological analysis of retrieved thrombotic material.

**Methods:**

In this monocentric exploratory study, clots from 200 patients undergoing MT were investigated by hematoxylin and eosin, CD3, and CD45 staining. Semiquantitative and computer-based image analysis defined the histological composition and relative fractions of immunohistochemically stained areas. First, we correlated these results with strokes of known etiology. Subsequently, clots of unknown source were characterized with regard to their (immuno)histological profile to attempt etiological classification.

**Results:**

Samples from 198 patients were accessible for analysis. Fibrin-/platelet-rich histology appeared in 45 (23%), erythrocyte-rich in 18 (9%), and mixed histology in 123 (62%) patients. Etiology was classified as cardioembolic in 87 (44%), arterioembolic in 37 (19%), and as cryptogenic stroke (CS) in 26 (13%) cases. 20 (23%) patients with cardioembolic stroke and 5 (14%) patients with arterioembolic stroke had fibrin-/platelet-rich clots. 8 (22%) patients with arterioembolic stroke and 1 (1%) patient with cardioembolic stroke had erythrocyte-rich clots. In CS, cardioembolic clot features appeared more than twice as often as arterioembolic clot features. Whereas the association between histology and etiology was significant (*p* = 0.0057), CD3/CD45 staining did not correlate.

**Conclusion:**

A significant association between histology and etiology was observed, with the proportion of erythrocyte-rich thrombi being largest among arterioembolic strokes and the proportion of fibrin-/platelet-rich thrombi highest among cardioembolic strokes. A high number of clots from CS presented histological features of cardioembolic clots. Thus, patients with CS and fibrin-/platelet-rich clots particularly require long-term cardiac rhythm monitoring and may benefit from oral anticoagulation.

**Supplementary Information:**

The online version of this article (10.1007/s10354-024-01060-w) contains supplementary material, which is available to authorized users.

## Introduction

Stroke etiology remains cryptogenic (cryptogenic stroke, CS) in up to 30% of patients with acute intracranial large vessel occlusion undergoing mechanical thrombectomy (MT) [1]. Patients with CS and complete diagnostic work-up and without competing etiologies are deemed to have suffered from an embolic stroke of undetermined source (ESUS). The results of the RESPECT and NAVIGATE ESUS trials that tested the efficacy of oral anticoagulation in ESUS do not support the long-held belief that most ESUS are predominantly caused by cardiac embolisms [2, 3]. Nevertheless, it is frequently hypothesized that a relevant portion of ESUS and CS are due to a cardiac origin. Prolonged cardiac rhythm monitoring via implantable loop recorders may disclose a cardioembolic (c.e.) pathology in many of these patients, with detection of atrial fibrillation in up to 23% of ESUS patients [4].

However, implantation of loop recorders in all ESUS patients would be too costly in most healthcare settings. Additional biomarkers would be helpful to narrow down the cohort of patients with a high probability of atrial fibrillation being diagnosed. Clinical trials such as ATTICUS and ARCADIA investigating secondary prevention strategies guided by various biomarkers of atrial cardiopathy (for example brain natriuretic peptide or suggestive electrocardiography parameters) did not find significantly reduced recurrent stroke risks with oral anticoagulation compared to antiplatelet therapy [5, 6].

Thrombus composition may constitute another innovative and promising marker to guide treatment decisions in secondary prevention for MT patients. Despite the increasing number of patients enrolled in studies using clot analyses, results are heterogeneous. While some trials failed to show a correlation between etiology and thrombus histology [7–9], others found erythrocyte-rich clots to be associated with cardioembolic stroke. However, unusually low recanalization rates, a small study population (37 patients), and exclusively postmortem analysis constitute major limitations of these studies [10–12]. Many authors found cardioembolic clots to be rich in fibrin, while others described clots from cryptogenic strokes to be fibrin rich and cardioembolic (a.e.) clots to be rich in red blood cells (RBC) [13–15]. Some groups detected a very similar histological pattern (fibrin and platelet predominance; F/P) in cardioembolic clots and those from ESUS patients [16, 17]. A meta-analysis in 2017 did not detect significant associations between clot histology and stroke etiology, while a more recent meta-analysis including 21 articles found that fibrin composition is significantly more common in strokes of cardioembolic and cryptogenic origin [18, 19]. Another recent retrospective analysis including 1350 patients showed arterioembolic thrombi to have a significantly higher mean RBC density and a lower platelet density than cardioembolic thrombi [20].

The association of F/P-rich white clots with cardioembolic and of RBC-rich red clots with an arterioembolic source seemingly contradicts long-traded concepts of hemostaseology and thrombogenesis rooted in the thoughts of Virchow at the beginning of the 20th century [21]. These concepts constitute that white clots originate in high-flow (e.g., carotid artery) and red clots in low-flow (e.g., left atrium appendage) areas. Current authors promote the pathophysiological hypothesis of clots forming at a ruptured plaque site (primarily in the carotid artery) consisting of a loose fibrin mesh which “catches” mainly erythrocytes and, hence, appears red [10, 22, 23]. This would imply that a.e. clots predominantly consist of RBC. Conversely, a clot developing in the left atrium appendage over a longer period of time displays a higher degree of fibrin polymerization and fibrin cross-linkages and, therefore, is richer in fibrin and more compact [24, 25]. Since our current pharmaceutical strategy for prevention of cardiac embolism in patients with atrial fibrillation ultimately consists of fibrin polymerization inhibition using non-vitamin-K oral anticoagulants, we would expect fibrin-rich clots in stroke patients with atrial fibrillation in the absence of an oral anticoagulant.

While inflammatory processes have for some time been known to play a key role in the development of arteriosclerosis, the relevance of inflammatory processes in acute vascular events—so-called immunothrombosis or thromboinflammation—has only recently received more attention [26–28]. Despite the obvious relevance, only a small number of studies have investigated the association between immunohistological clot features and stroke etiology so far. No correlation between clot staining for CD68/KiM1P (macrophages), T and B cells (CD3, CD4, CD20), and CD68+ macrophages and stroke etiology has been found [16, 29]. In one study, the staining signal for citrullinated histone (displaying neutrophil extracellular traps) was significantly more abundant in cardioembolic and in more matured clots [30]. Dargazanli et al. [31] found that the number of CD3+ T cells was significantly higher in arterioembolic clots. However, low patient numbers [45] and manual quantification of staining intensity constitute limitations of this study [31].

The overarching goal of our study is to identify those CS patients with clot characteristics suggestive of cardiac embolism within a cohort of MT patients. This subgroup would be predestined for intensified electrocardiography (ECG) monitoring and—in case of detection of atrial fibrillation—anticoagulation.

We analyzed thrombotic material retrieved during MT on a histological and immunohistochemical (CD3, CD45) level in a large number of consecutive patients presenting to our MT center. CD3 is considered a pan T cell antigen. T cells are known to play an important role in the development of arteriosclerosis and have previously been described to be associated with arterioembolic strokes [31, 32]. CD45 is a more general marker of receptor-dependent leucocyte activation and is therefore crucially involved in the initiation and regulation of T cell receptor signaling [33]. We expected both markers to show a higher degree of staining intensity in clots from arterioembolic strokes compared to cardioembolic strokes due to ongoing inflammatory processes in arteriosclerosis.

## Materials and methods

A total of 200 adult stroke patients consecutively undergoing endovascular recanalization therapy at the Neurovascular Centre of Kepler University Hospital Linz (Neuromed Campus) between January 2018 and February 2019 were included in our study.

All clots were immediately fixed in 4% formaldehyde and embedded in paraffin. 4 μm thick sections from the level of maximum spatial expansion of the clot were used for further analysis.

The site of slice sampling was chosen on the basis of a theory of clot formation that suggests two components: the primary thrombus (which would be the relevant material for etiological analyses) and the secondary thrombus that forms due to blood stasis around the primary one [34]. We assumed that at the site of maximum expansion, the chances of retrieving primary thrombus would be highest.

**Fig. 1:**
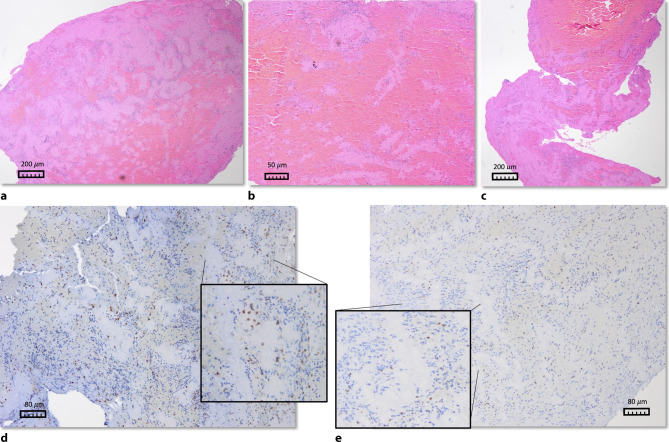
**a**–**c** H&E-stained clots: **a** mixed clot, four-times magnification; **b** erythrocyte-rich clot, 16-times magnification;** c** separated clot with erythrocyte-rich fraction in the upper region and fibrin-/platelet-rich fraction in the lower region, four-times magnification. **d **CD45-stained clot with intense staining signal, 10-times magnification. **e** CD3-stained clot with moderate staining signal, 10-times magnification

Consecutive slides of a clot were stained for hematoxylin and eosin (H&E) as well as immunohistochemically for CD3 and CD45 (pretreatment in citrate buffer at pH 6; CD3: Thermo Scientific, dilution 1:100; CD45 Dako, dilution 1:800). All slices were analyzed at a microscope (Olympus, BX53) with 10-times objective magnification, photographed in digital form (Olympus, SC50; resolution 300 dpi), and rated visually. H&E-stained samples were classified according to the fractions of their main components (F/P, RBC) as previously described [17]. Samples consisting of more than 80% F/P or RBC were assigned to one of the groups F/P- or RBC-rich; otherwise they were classified as mixed clots (Fig. 1). The fraction of immunohistochemically stained cells was visually rated on a scale from 0 to 2 in 0.5 steps, where 0 displayed no cell stained and 2 the most intense staining appearing in the whole group of samples. Visual analysis was performed after intensive training of the first author of this article D. C. Schwarzenhofer at the Institute of Neuropathology of Kepler University Clinic in Linz, Austria, and under strict observation by a  enior neuropathologist. All raters were blind to clinical and interventional data. In cases of interobserver discrepancies, another experienced neuropathologist was consulted (further methodological details provided in Supplemental I).

**Fig. 2:**
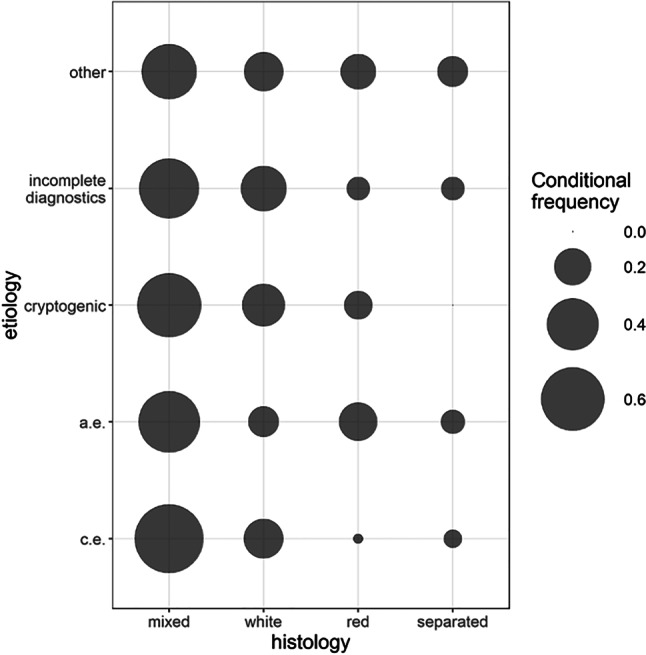
Histological clot characteristics and stroke etiology (*c.e.* cardioembolic, *a.e.* arterioembolic, *white* fibrin-/platelet-rich, *red* erythrocyte-rich, *separated* two clearly divided red and white sections within one clot—mean used)

Semi-automated analysis calculating the relative fraction of immunohistologically stained cells was performed by taking photographs from a representative section of the slide (after microscopic inspection of the whole slide) and application of the ImageJ software (ImageJ 1.52v; Rasband, W. S., U. S. National Institutes of Health, Bethesda, Maryland, USA, https://imagej.nih.gov/ij/, 1997–2018). H. E.-stained slides were rated visually only, immunohistologically stained slides were rated visually and analyzed semi-automatically. In a few cases, thrombotic material presented with a high degree of intra-thrombus variability concerning its main (immuno)histological components, so a single representative section could not be chosen. In these cases, the histology was classified as “separated” (Table 2; Fig. 2) and for further immunohistochemical analysis, two distinctive sections were rated separately and the mean of the two values was used (consort flow diagram Supplementary Fig. 3, Supplemental III).

Subsequently, a broad set of demographic, clinical, interventional, and imaging data was collected from the electronic patient record (SAP; KUK_KIS) and the Austrian Stroke Unit Registry (for details see Supplemental I). Stroke etiology was determined by the TOAST classification, with an additional group for patients with incomplete diagnostics [35]. Cardioembolic stroke was defined as stroke with a high-risk cardioembolic source (presence of either atrial fibrillation, a mechanical valve or thrombus in the left ventricle/atrium/atrial appendage, mitral stenosis with atrial fibrillation, atrial fibrillation [other than lone atrial fibrillation], sick sinus syndrome, recent myocardial infarction [< 4 weeks], dilated cardiomyopathy, akinetic left ventricular segment, atrial myxoma, or infective endocarditis), and arterioembolic stroke was defined as stroke in the vascular territory of an internal carotid artery displaying an occlusion of > 50%. Diagnosis of CS required completion of at least transthoracic echocardiography, 24-hour electrocardiography, and carotid artery duplex scan. For detailed information on semi-automated image analysis, etiological classification, and the elicited data, please refer to Supplemental I.

For statistical analysis absolute and relative frequencies were computed for nominal variables. Metric non-normal and ordinal variables are presented with median and interquartile range (IQR). Normality of the variables was tested using the Shapiro–Wilk test. Association between nominal variables such as etiology and histology or adjusted mRS at onset and etiology was tested using Fisher’s exact test. For nominal variables with many categories, *p*-values of Fisher’s exact tests were computed by Monte Carlo simulation using 100,000 simulations. Kruskal–Wallis tests were performed to test for differences in metric variables between more than two independent groups, such as in the distribution of semi-automatically rated CD3 and CD45 by categories of etiology or in the distribution of NIHSS at onset by categories of etiology. Since only few subjects had mRS values of 0, 1, or 2, a variable “adjusted mRS” was created with values 0–2, 3, 4, and 5. Spearman’s correlation coefficient was calculated for the association between semi-automatically and visually rated CD3/CD45 and for the association between semi-automatically rated CD3/CD45 and time to recanalization. Mann–Whitney U test was used to test for differences between semi-automatically rated CD3/CD45 staining intensity between gender groups.

A log-rank test was performed to test for differences in the hazard rates of time to recanalization across histological groups. The association between histology and etiology is visualized by bubble plots. The distribution of semi-automatically analyzed CD3 staining intensity by clot histology is shown in boxplots. The level of significance was set to 0.05.

First, we investigated the relationship between (immuno)histological clot characteristics and stroke etiology in a subgroup of patients with known etiologies. Subsequently, we investigated clot characteristics of CS with the aim of matching them with the (immuno)histological patterns defined in the first cohort. Our study was designed as an exploratory trial.

## Results

A total of 200 clots were collected, and 198 clots were of sufficient quality for further analysis (two were excluded due to a very high degree of fragmentation). The most relevant demographic and clinical characteristics of our study population, (immuno)histological clot characteristics, and stroke etiology are provided in Tables 1, 2, and 3; Fig. 2.

**Table 1:** Main demographic and clinical characteristics of acute stroke patients undergoing MT

Characteristics	*n* = 198
Female, *n* (%)	89 (45)
Age, years, median (IQR)	71 (59–80)
Previous vascular event, yes (%)	58 (29)
M1 occlusion, *n* (%)^a^	111 (56)
M2 occlusion, *n* (%)^a^	32 (16)
M3 occlusion, *n* (%)^a^	1 (0.5)
A1 occlusion, *n* (%)^a^	3 (1.5)
Carotid T occlusion, *n* (%)^a^	35 (18)
Distal ICA occlusion, *n* (%)^a^	43 (22)
Proximal ICA occlusion, *n* (%)^a^	32 (16)
CCA, *n* (%)^a^	4 (2)
Basilar artery, *n* (%)^a^	23 (12)
Vertebral artery, *n* (%)^a^	9 (4.5)
PCA, *n* (%)^a^	4 (2)
Side, left *n* (%), right *n* (%)	98 (49), 78 (39)
Thrombolysis, yes, *n* (%)	124 (63)
TICI 3, *n* (%)	137 (69)
TICI 2c, *n* (%)	26 (13)
TICI 2b, *n* (%)	21 (11)
TICI 2a, *n* (%)	1 (0.5)
TICI 1, *n* (%)	1 (0.5)
TICI 0, *n* (%)	2 (1)
TICI no information, *n* (%)	10 (5)
NIHSS onset, median (IQR)	15 (11–18)
NIHSS improvement, median (IQR)	10 (5–14)
Death, *n* (%)	40 (20)

*M1/2/3 occlusion* occlusion of the M1/2/3 segment of the middle cerebral artery, *A1 occlusion* occlusion of the anterior cerebral artery, *ICA* internal carotid artery, *CCA* common carotid artery, *PCA* posterior cerebral artery, *thrombolysis* intravenous bridging thrombolysis, *previous vascular event* stroke, myocardial infarction (> 4 weeks), pulmonary embolism, deep venous thrombosis, occlusive peripheral arterial disease with interventional treatment, *TICI* Thrombolysis in Cerebral Infarction, *NIHSS* National Institute of Health Stroke Scale

^a^Several patients suffered from more than one vessel occlusion

**Table 2:** Histological clot characteristics and stroke etiology

Histology			Etiology			
	c.e.	a.e.	Cryptogenic	Incomplete	Other	Total
Mixed (*n*)	62	21	16	14	10	123
F/P-rich (*n*)	20	5	7	8	5	45
RBC-rich (*n*)	1	8	3	2	4	18
Separated (*n*)	4	3	0	2	3	12
Total (*n*)	87	37	26	26	22	198

*c.e.* cardioembolic, *a.e.* arterioembolic, *incomplete* incomplete diagnostics, *RBC* red blood cells, *F/P* fibrin/platelet, *separated* two clearly divided red and white sections within one clot

**Table 3:** Distribution of clot (immuno)histology and stroke etiology

	Cardioembolic	Arterioembolic	Cryptogenic	*p*-value
RBC-rich (%)	1	22	11	–
F/P-rich (%)	23	13	27	–
Mixed (%)	71	57	62	–
Separated (%)	5	8	0	0.0057
Sum (%)	100	100	100	–
CD3 visually (0-2)^a^	0.5	0.5	0.5	0.8608
CD45 visually (0–2)^b^	1.5	1.5	1/1.5	0.3806
CD3 semi-automated, median	0.12	0.11	0.14	0.4732
(IQR)	(0.04–0.26)	(0.07–0.2)	(0.09–0.21)	–
CD45 semi-automated, median	0.39	0.4	0.41	0.4395
(IQR)	(0.25–0.6)	(0.23–0.52)	(0.26–0.5)	–

*RBC* red blood cells, *F/P* fibrin/platelet, *separated* two clearly divided red and white sections within one clot—mean used,

^a^Most prevalent value on a range from 0 to 2 in 0.5 steps

^b^For greater clarity, tabular presentation is restricted to the three most relevant etiological groups

Significant associations were observed between histology and etiology (*p* = 0.0057) considering all etiological groups including CS but also when restricting the analysis to the two etiological groups of cardio- and arterioembolic strokes (*p* < 0.001). RBC-rich (“red”) clots were observed more often in arterioembolic strokes, while F/P-rich (“white”) clots were clearly more apparent in cardioembolic strokes. Only 26 (13%) of all patients had CS; of these, 16 (62%) had mixed, 7 (27%) F/P-rich, and 3 (11%) RBC-rich clot histology.

No significant association was observed between immune staining and different etiological groups, neither in semi-automated nor in visual analysis. Spearman’s correlation coefficient for semi-automated and visual CD3 ratings was 0.8513 and was 0.818 for semi-automated and visual CD45 ratings (*p*-values < 0.001 for both tests; Supplementary Fig. 2, Supplemental III).

The distribution of CD3 staining intensity varied significantly between different histological groups, with most intense staining observed for mixed clots (*p* = 0.0026; Supplementary Fig. 1, Supplemental III; Supplementary Table 3, Supplemental II). Compared to F/P-rich clots, RBC-rich clots showed a tendency towards more intense CD45 staining (*p* = 0.0542).

Application of thrombolytic therapy was not significantly associated with the results of immunohistochemical staining but was associated with histological patterns. The association observed for etiology and application of thrombolysis was also significant (*p* = 0.0483; Table 4). A higher relative fraction of F/P-rich clots in cardioembolic strokes was observed in patients without thrombolysis compared to patients with thrombolysis (c.e. stroke: thrombolysis—clot: mixed 78%, F/P-rich 17%, RBC-rich 2%, separated 3%; c.e. stroke: no thrombolysis—clot: mixed 57%, F/P-rich 36%, RBC-rich 0%, separated 7%).

**Table 4:** Distribution of clinical and interventional parameters, clot (immuno)histology and stroke etiology

	RBC	F/*P*	Mixed	*p*-value	CD3 *p*-value	CD45 *p*-value	c.e.	a.e.	Crypt	*p*-value
Thrombolysis, yes (%)	76	56	68	0.0483	0.6448	0.7798	67	50	85	0.0015
Age, years, median	60	73	73	0.0131	0.0629	0.9763	79	64	70	< 0.001
(IQR)	(56–67)	(58–82)	(64–81)				(70–86)	(60–71)	(61–78)	
No. occluded vessels, median	2	1	1	0.0088	0.8067	0.4524	1	2	1	< 0.001
(IQR)	(1–2)	(1–1)	(1–2)				(1–1)	(1–2)	(1–2)	
TICI 3 (%)	83	57	74	0.1522	0.8607	0.6826	72	82	62	0.5288
Time to final recanalization, min, median	244	254	245	0.9341	0.3477	0.0096–0.277^a^	256	226	256	0.3870
(IQR)^b^	(222–324)	(235–293)	(206–311)				(209–297)	(214–249)	(199–320)	

*thrombolysis* intravenous bridging thrombolysis, *RBC* red blood cells, *F/P* fibrin/platelet, *CD3/CD45* semi-automated, *c.e.* cardioembolic, *a.e.* arterioembolic, *crypt.* cryptogenic stroke

^a^Correlation coefficient, For greater clarity, tabular presentation is restricted to the three most relevant etiological groups

^b^Time to final recanalization from stroke onset, *no.* number

A significant association was observed between patient age and histological patterns as well as between age and etiological groups (Table 4). No significant correlation was observed between immune staining results and age of the patients (Table 4).

A significant difference was observed between the number of occluded vessel sections and clot histology (Supplementary Table 2, Supplemental II) but not for semi-automated immunohistochemical staining results (CD3 *p* = 0.8067; CD45 *p* = 0.4524).

With regard to interventional parameters, the proportion of patients in whom TICI 3 was achieved was higher in patients with red clots compared to white clots (Table 4). However, differences between TICI score and (immuno)histology or stroke etiology were not significant (Table 4).

Spearman’s correlation coefficient between CD45 (semi-automated analysis) and time to recanalization was significant at −0.2777 (*p* = 0.0096), i.e., a longer period between stroke onset and final vessel recanalization was associated with lower CD45 staining intensity (Table 4). Other possible confounders had no significant association with (immuno)histological characteristics (Supplementary Table 1, Supplemental II).

## Discussion

Our study aimed to investigate the association between histological and immunohistochemical characteristics of clots retrieved in MT with different stroke etiologies. Except for a lower proportion of CS (13%), demographic and clinical characteristics of our cohort matched well with those of previously published MT cohorts. The discrepancy concerning CS may reflect more frequent loop recorder implantations, effects of sample size, and the single-center study design [1, 17].

The key question of this study, “Does clot composition permit to draw conclusions about stroke etiology?” can be affirmed in terms of histological analysis. For one third of our patients a significant association between histological clot subtype and etiology (“F/P-rich”—cardioembolic, “RBC-rich”—arterioembolic) was observed. These results are in line with those obtained in larger cohorts than ours [18–20]. This underlines the validity of our approach. Hence, we recommend routine microscopic processing of thrombi retrieved during MT to aid etiological classification. This may include standardized histological pattern analysis, which has been described as a promising approach towards more precise etiological determination [36].

Possible confounders hampering the application of histological clot analysis for diagnostic purposes include the effects of recanalization therapies and the presence of a mixed clot composition. In two thirds of our patients, a mixed clot histology was detected. Mixed clots are hypothesized to be driven mainly by appositional thrombotic material. This, in turn, has been shown to be strongly dependent on angio-architecture and collaterals [37]. Therefore, the question arises of whether the sections selected for analysis in mixed clots are representative of the primary clot. Previous research is ambiguous on this issue: on the one hand, low intra-thrombus variability concerning the predominant histological components has been shown by sectioning multiple whole thrombi, but on the other hand, heterogeneity in histological clot composition during different passages of MT has been described [34, 38]. Partial sectioning of the thrombus has been described to give a good estimate of the overall histological thrombus composition and is recommended [38]. In our study, all thrombotic material retrieved during MT was visually assessed and cases with high intra-thrombus variability were taken into account in the histological group “separated” and by calculating the mean of two sections with clearly different immunohistochemical staining intensity.

Patients with white clots had received acute intravenous thrombolytic therapy in fewer cases than patients with other clot histology, although patients with cardioembolic strokes received thrombolysis more often than patients with arterioembolic strokes. The effect of fibrin depolymerization during thrombolysis leading to a lower fibrin content in clots has previously been described [34, 39, 40]. We assume that application of thrombolysis masks the original fibrin content and potentially shifts originally F/P-rich (c.e.) clots towards mixed clots. Consequently, the association between white clots and cardioembolic etiology was less altered in the c.e. strokes which were not treated with thrombolysis. This hypothesis is supported by the observation of a higher fraction of F/P-rich clots in c.e. strokes in patients without thrombolysis in our cohort. In those patients with clearly F/P-rich clots despite application of thrombolysis, there may have been a relative resistance of the thrombus to thrombolysis, e.g., due to a long appositional thrombus.

The association between lower patient age and RBC-rich clots is most likely driven by lower age in patients with arterioembolic strokes [41].

We did not observe differences in CD3 and CD45 staining intensity between different stroke etiologies in our cohort. In particular, previous findings of an association between CD3 staining and arterioembolic stroke could not be replicated. Considering the well-balanced subgroups and the large sample size of our cohort, we do not assume methodological biases to have contributed to the negative results, though visual selection of the most representative clot section could theoretically have influenced the results. Visual and semi-automated slide ratings were highly correlated, indicating the reliability of both processes.

While CD45 and CD3 staining intensity did not directly correlate with etiology, we did observe a higher CD45 staining signal in red clots than in white clots (Supplementary Table 3, Supplemental II). This may indirectly reflect inflammatory processes in arteriosclerosis and thrombogenesis.

Since a significant association was observed between the duration of vessel occlusion and a decreasing CD45 signal, negative immunohistochemical study results could potentially be explained by a process of consumption of T cells in the very early phase after vessel occlusion, followed by a shift in immune response towards other, not yet detected, cellular or humoral components [42]. Thus, we propose that thromboinflammation constitutes an important pathophysiological process in the genesis of ischemic stroke. This is in line with previous reports [28]. However, future studies may have to stratify results on CD45 staining according to the time from stroke onset to the time of first MT passage. Alternatively, immunohistochemical markers only staining the primary clot or immunological components that are present in later stages of the immunological cascade may be more promising candidates for immunohistochemical detection of clot origin. These include H3Cit (neutrophil extracellular traps), CD11c and CD68 (macrophage activity), CD66b and CD15 (neutrophilic leukocyte), CD106 (vascular endothelium), PECAM‑1 (platelet endothelial cell adhesion molecule), CD34 (endothelial cells), and glycoprotein VI (platelet signal-transducing receptor) [30, 43–45]. Further investigations should also be steered towards identifying a specific marker for cardiac endothelium.

Our study has several limitations: staining for H&E does not allow a sufficient distinguish between fibrin and platelets, which could potentially provide a more precise clot characterization in a higher number of patients. Thrombolysis, physical forces during MT, and the choice of the applied device have been described to influence clot composition [40]. The low number of CS patients in our cohort poses a limitation, and categorical histological assignment could have led to loss of information due to high cut-off values (80%). A mixed clot histology detected in two thirds of our patients further limits the broad clinical applicability of conventional histological analysis to determine stroke etiology.

## Conclusion

In our study we observed a significant association between clot histology and stroke etiology. The strong overlap of the histological characteristics of cardioembolic clots and those from CS patients supports the pathophysiological hypothesis of cardiac embolism in most CS patients. Standardization in histological staining, clot evaluation, and terminology may further strengthen the approach of histological clot examination. CD3 and CD45 staining intensity did not correlate with stroke etiology. We propose modifications of methodology and alternative immunological markers that future studies may employ in order to prove the significance of thromboinflammation for etiological classification of stroke.

The findings of this study may justify a prospective treatment trial with direct oral anticoagulants (DOACs) in patients with ESUS and F/P-rich clot histology. Oral anticoagulation outside of clinical studies merely on the basis of clot histology does not seem reasonable at this point. More importantly, the evaluation process for implantable loop recorders in ESUS could also be guided by clot histology in future clinical routine, since their use in all ESUS patients would be too costly in most healthcare settings.

## Supplementary Information

Supplemental Files I

Supplemental Files II

Supplemental Files III

## Data Availability

The main author has full access to all data in the study and takes responsibility for the study’s integrity and the data analysis.

## Abbreviations

a.e.: Arterioembolic
c.e.: Cardioembolic
CARE: Configuration analysis to refine etiology
CD: Cluster of differentiation
CS: Cryptogenic stroke
DOACs: Direct oral anticoagulants
ESUS: Embolic strokes of undetermined source
F/P: Fibrin/platelet
MT: Mechanical thrombectomy
PAI: Platelet aggregation inhibition
RBC: Red blood cells
TICI: Thrombolysis in cerebral infarction
