# Supplementary material for: Thrombo-CARE—cardioembolic stroke etiology in cryptogenic stroke suggested by fibrin-/platelet-rich clot histology: Thrombo-CARE (configuration analysis to refine etiology)
Source: Wien Med Wochenschr. 2024 Nov 11;175(9-10):227–36. doi: 10.1007/s10354-024-01060-w (PMC12089195; doi:10.1007/s10354-024-01060-w)
Supplement: Supplementary file 1 — Supplemental Files I [file 10354_2024_1060_MOESM1_ESM.docx]

**Supplemental I - Thrombo-CARE study:**

**Methods**:

Clinical data:

The data set included information on sex, age, National Institute of Health Stroke Scale (NIHSS) and modified Rankin Scale (mRS) at onset and discharge, wake-up stroke vs. observed stroke onset, previous vascular events (stroke, myocardial infarction, pulmonary embolism, deep venous thrombosis, occlusive peripheral arterial disease with interventional treatment), active malignancy (diagnosis of tumor or non-remission of tumor within the last five years), premedication (platelet aggregation inhibition (PAI), (direct) oral anticoagulation ((D)OAC)), effect of (D)OAC (OAC: International Normalized Ratio (INR)/prothrombin time, DOAC: Anti-Xa activity/thrombin time or/and serological drug level), atrial fibrillation (previously known, not or newly detected), detection of patent foramen ovale, occluded vessel(s), application of thrombolytic therapy, time to thrombolysis and recanalization by MT, applied thrombectomy device, duration of intervention (aspiration catheter and/or stent retriever), number of catheter passages, recanalization score (Thrombolysis in Cerebral Infarction (TICI)), complications (symptomatic intracranial bleeding or cerebral edema) and death (less than three months from hospital discharge). No distinction was made between anterior and posterior circulation or section of vessel occlusion (for example intracranial ICA or M1 occlusion) for analysis.

Stroke etiology was determined by TOAST classification, with an additional group for patients with incomplete diagnostics [1]. Cryptogenic stroke also includes patients with two or more given potential stroke sources (this applied to 3 patients in our cohort) and patients with incomplete diagnostic [2]. In our study the latter were excluded from the group of cryptogenic stroke and categorized as “incomplete” in a separate group, thus allowing diagnosis of ESUS in most of our patients with cryptogenic stroke and to avoid bias towards factors that led to incomplete diagnostics. These factors constitute early death in 68% but also include aspects such as place of residence, partly resulting in omission of further diagnostics in peripheral hospitals after return transfer. Due to 3 patients with two potential stroke sources and to avoid confusion for the reader, the less specific term “cryptogenic stroke” was used for all patients with complete diagnostic work-up and unclear stroke etiology, though most of them fulfilled criteria for ESUS.

Patients from the etiological group “other determined etiology” (other) mainly suffered from dissections, paradoxical embolism or paraneoplastic thromboembolism.

Staining and image analysis:

As hematoxylin and eosin staining does not allow to adequately distinguish fibrin and platelets, the histological subgroup “F/P-rich” was established, as previously applied in several studies on this field [3-7]. Analyzing one slide per staining per clot follows recent recommendations [8]. The cut-off value of 80% to distinguish histological subgroups was set under consideration of a previous study, describing a receiver operating characteristic analysis with a selected cutoff value of 60% F/P content resulting in a positive predictive value of 80.7% for distinguishing cardioembolic stroke [3]. The value of 80% was set to further improve positive predictive value of clot histology on stroke etiology and due to established standards of our Institute of Pathology concerning this matter.

The semi-automated image analysis process consisted of a deconvolution of the different colors in the slides, counterstaining of the brownish parts (reflecting immunohistochemical staining signal) into red, depending on an individual, manually set threshold of signal intensity and, if the chosen section did not fill the whole image frame, manually cutting out the clot. Finally, the relative fraction of areas positive for immunohistochemical staining was calculated.

Statistical analysis:

To investigate the difference in thrombus composition comparing patients with and without previous thrombolysis multivariate analysis was intended in order to exclude the possible influence of this treatment on the association between thrombus composition and different pathogenic subtypes of ischemic stroke. Due to the small number of patients in individual subgroups, multivariate analysis was not feasible and an analysis of the bivariate distribution and association of histology and etiology in the subgroups of patients with and without thrombolysis was performed instead. Only five patients in our cohort had known active malignancy, due to this small number no statistical tests were performed.

1. Adams HP, Bendixen BH, Kappelle LJ, Biller J, Love BB, Gordon DL, et al. Classification of subtype of acute ischemic stroke. Definitions for use in a multicenter clinical trial. TOAST. Trial of Org 10172 in Acute Stroke Treatment. Stroke. 1993;24(1):35-41. doi: 10.1161/01.str.24.1.35.

2. Ntaios G. Embolic Stroke of Undetermined Source: JACC Review Topic of the Week. J Am Coll Cardiol. 2020;75(3):333-40. doi: 10.1016/j.jacc.2019.11.024.

3. Boeckh-Behrens T, Kleine JF, Zimmer C, Neff F, Scheipl F, Pelisek J, et al. Thrombus Histology Suggests Cardioembolic Cause in Cryptogenic Stroke. Stroke. 2016;47(7):1864-71. doi: 10.1161/STROKEAHA.116.013105.

4. Marder VJ, Chute DJ, Starkman S, Abolian AM, Kidwell C, Liebeskind D, et al. Analysis of thrombi retrieved from cerebral arteries of patients with acute ischemic stroke. Stroke. 2006;37(8):2086-93. doi: 10.1161/01.STR.0000230307.03438.94.

5. Kim SK, Yoon W, Kim TS, Kim HS, Heo TW, Park MS. Histologic Analysis of Retrieved Clots in Acute Ischemic Stroke: Correlation with Stroke Etiology and Gradient-Echo MRI. AJNR Am J Neuroradiol. 2015;36(9):1756-62. doi: 10.3174/ajnr.A4402.

6. Choi MH, Park GH, Lee JS, Lee SE, Lee SJ, Kim JH, et al. Erythrocyte Fraction Within Retrieved Thrombi Contributes to Thrombolytic Response in Acute Ischemic Stroke. Stroke. 2018;49(3):652-9. doi: 10.1161/STROKEAHA.117.019138.

7. Brinjikji W, Duffy S, Burrows A, Hacke W, Liebeskind D, Majoie CBLM, et al. Correlation of imaging and histopathology of thrombi in acute ischemic stroke with etiology and outcome: a systematic review. J Neurointerv Surg. 2017;9(6):529-34. doi: 10.1136/neurintsurg-2016-012391.

8. Staessens S, Fitzgerald S, Andersson T, Clarençon F, Denorme F, Gounis MJ, et al. Histological stroke clot analysis after thrombectomy: Technical aspects and recommendations. Int J Stroke. 2020;15(5):467-76. doi: 10.1177/1747493019884527.
