# Supplementary material for: Thrombo-CARE—cardioembolic stroke etiology in cryptogenic stroke suggested by fibrin-/platelet-rich clot histology: Thrombo-CARE (configuration analysis to refine etiology)
Source: Wien Med Wochenschr. 2024 Nov 11;175(9-10):227–36. doi: 10.1007/s10354-024-01060-w (PMC12089195; doi:10.1007/s10354-024-01060-w)

**Supplemental II - Thrombo-CARE study:**

**Supplementary Table 1.** Negatively tested potential confounders for (immuno-)histological clot composition.

| **variable** | **p-value** for **histology/CD3/CD45** |
| --- | --- |
| gender | 0.6102/0.2929/0.2539 |
| premedication | 0.0668/0.5908/0.7681 |
| vessel dissection | 0.2571/0.2305/0.5102 |
| duration of thrombectomy | 0.7151/0.5137/0.2201 |
| number of catheter passages | 0.1031/ 0.1133/0.3277 |
| time to final vessel recanalization | 0.9341/0.3477; CD45: see results |
| time from clinical onset to  application of thrombolysis | 0.6154/0.7427/0.5287 |

**Supplementary Table 2.** Analysis of histology and number of occluded vessel sections (white = fibrin/platelet-rich; red = erythrocyte-rich; separated = two clearly divided red and white sections within one clot – mean used; nr. = number of occluded vessel sections; p = 0.0088).


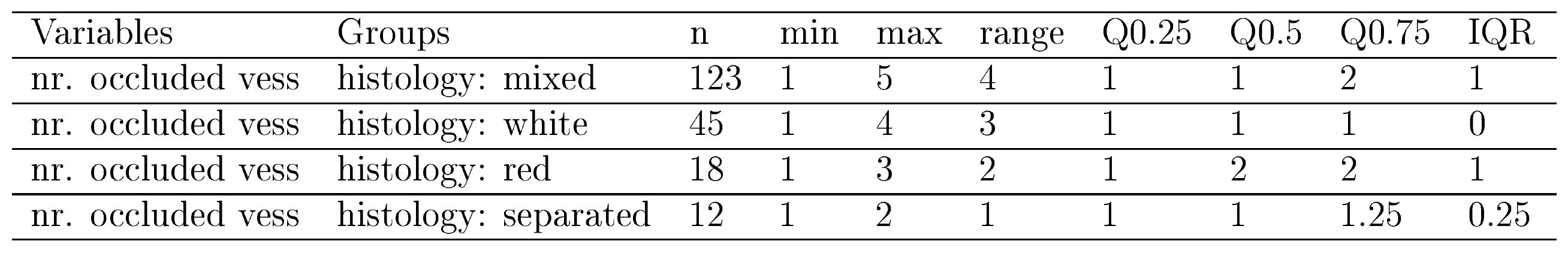


**Supplementary Table 3.** Analysis of semi-automated rated CD3- and CD45-staining intensity and histology (separated = two clearly divided red and white sections within one clot – mean used; CD3 p = 0.0026; CD 45 p = 0.1294).


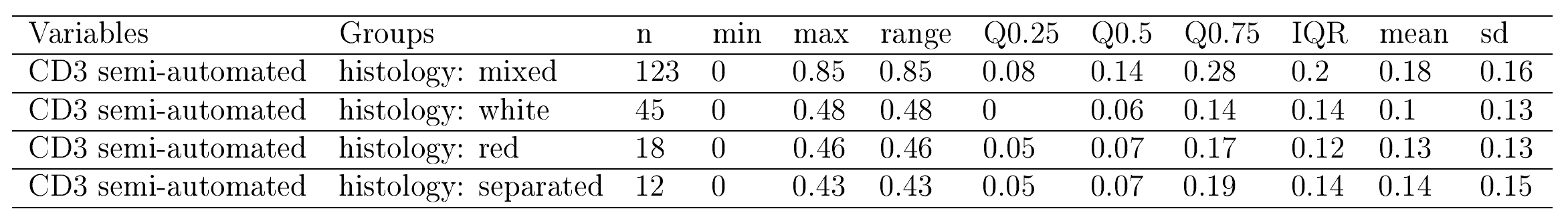


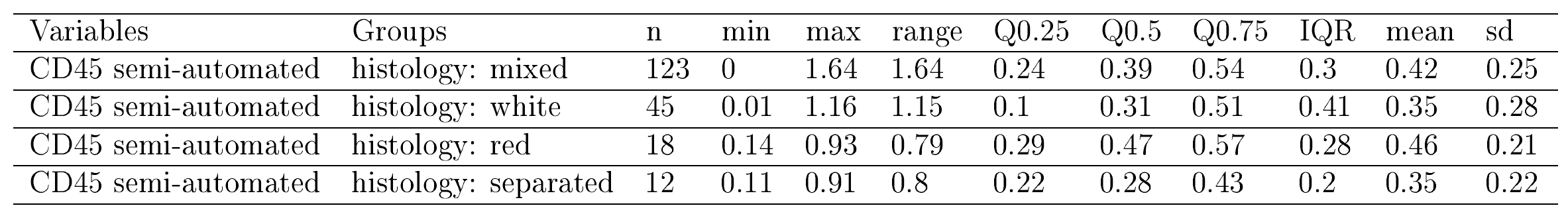

Supplement: Supplementary file 2 — Supplemental Files II [file 10354_2024_1060_MOESM2_ESM.docx]
