# Supplementary material for: Thrombo-CARE—cardioembolic stroke etiology in cryptogenic stroke suggested by fibrin-/platelet-rich clot histology: Thrombo-CARE (configuration analysis to refine etiology)
Source: Wien Med Wochenschr. 2024 Nov 11;175(9-10):227–36. doi: 10.1007/s10354-024-01060-w (PMC12089195; doi:10.1007/s10354-024-01060-w)
Supplement: Supplementary file 3 — Supplemental Files III [file 10354_2024_1060_MOESM3_ESM.docx]

**Supplemental III - Thrombo-CARE study:**

**Supplementary Figure 1.** Semi-automated rated CD3 staining (%) by clot histology (white = fibrin/platelet-rich; red = erythrocyte-rich; separated = two clearly divided red and white sections within one clot – mean used).

**
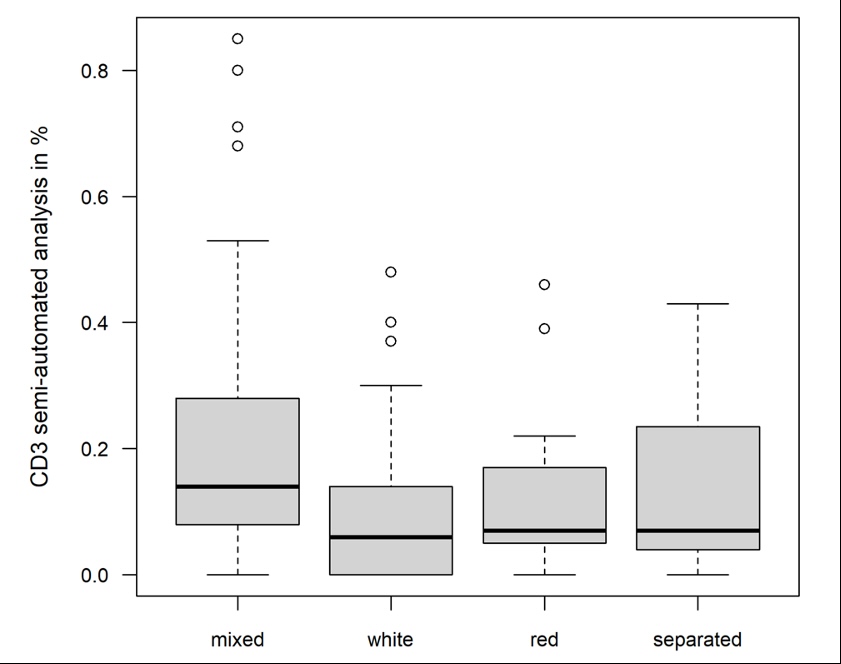
**

**Supplementary Figure 2.** Correlation between visual and semi-automated rated CD3- and CD45-staining intensity (visual ratings on scale from 0-2 in 0.5 steps, semi-automated ratings of fraction of immunohistochemical stained cells (%); correlation coefficient CD3 0.8513; correlation coefficient CD 45 0.818; p < 0.001).


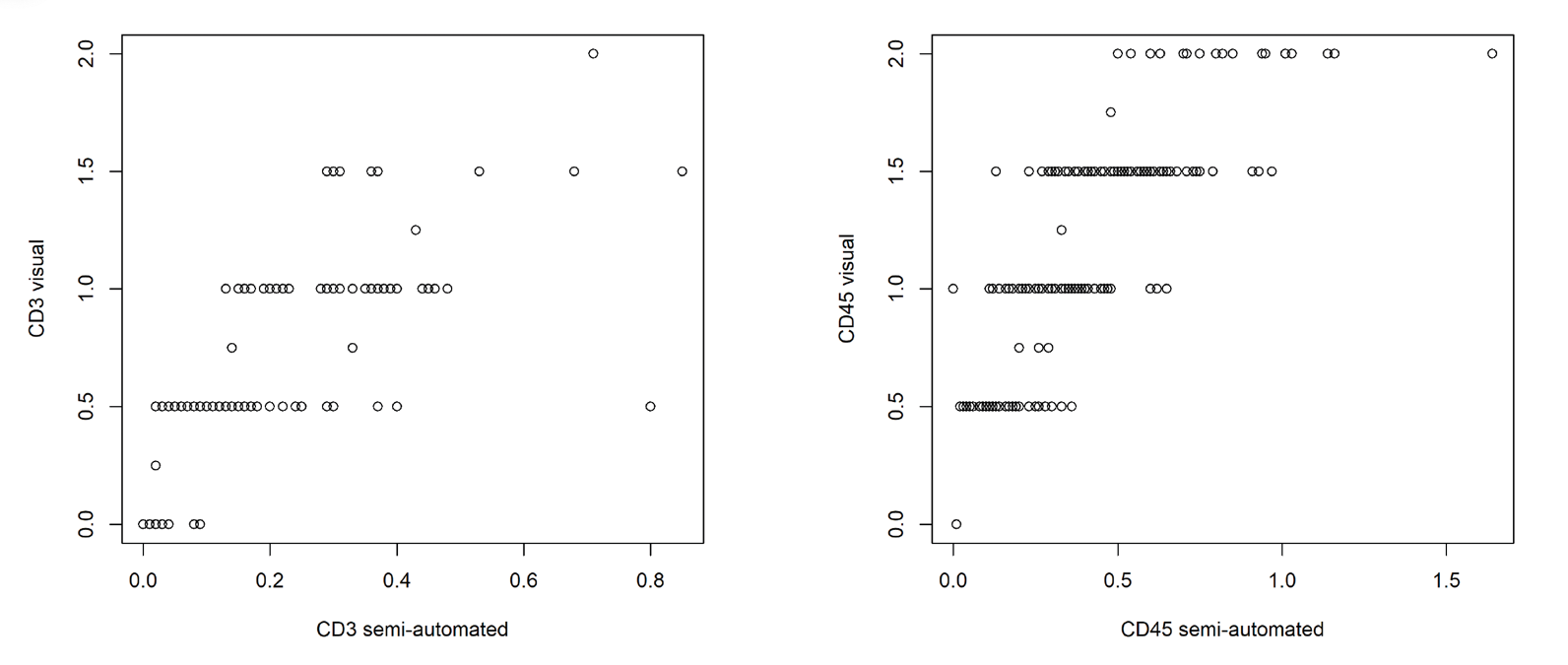


**Supplementary Figure 3.** Consort-Diagram (RBC = red blood cells; F/P = Fibrin/Platelet; separated = two clearly divided red and white sections within one clot – mean used; *most prevalent value on a range from 0-2 in 0.5 steps)

**CONSORT Flow Diagram Thrombo-CARE study**

arterio-embolic

0.11 (0.07 – 0.2)

0.12

(0.04 – 0.26)

semi-automated analysis

staining intensity per etiological group (median, IQR)

cardio-embolic

0.5/ 1.5

0.12

(0.04 – 0.26)

arterio-embolic

0.5/ 1.5

0.12

(0.04 – 0.26)

cryptogenic

0.5/ 1/1.5

0.12

(0.04 – 0.26)

cryptogenic

0.14 (0.09 – 0.21)

0.12

(0.04 – 0.26)

cardio-embolic

0.12 (0.04 – 0.26)

0.12

(0.04 – 0.26)

separated (n=12)

F/P-rich (n=45)

RBC-rich (n=18)

mixed

(n=123)

Histological analysis (n=198)

(hematoxylin and eosin staining)

Analyzed (n=198)

Assessed for eligibility (n=200)

Excluded (n=2)

♦  to high fragmentation of thrombotic material (n=2)

Immunohistological analysis (n=198)

(CD3- and CD45 staining)

## Analysis

## Results

visual analysis

staining intensity per etiological group (0 - 2*; CD3/CD45)

## Enrollment
